# Correction to: The antioxidant Trolox restores mitochondrial membrane potential and Ca^2+^-stimulated ATP production in human complex I deficiency

**DOI:** 10.1007/s00109-021-02057-3

**Published:** 2021-06-10

**Authors:** Felix Distelmaier, Henk-Jan Visch, Jan A. M. Smeitink, Ertan Mayatepek, Werner J. H. Koopman, Peter H. G. M. Willems

**Affiliations:** 1 Department of Biochemistry (286), Nijmegen Centre for Molecular Life Sciences, Radboud University Nijmegen Medical Centre, P.O. Box 9101, 6500 HB Nijmegen, The Netherlands; 2 Department of Pediatrics, Nijmegen Centre for Mitochondrial Disorders, Radboud University Nijmegen Medical Centre, Nijmegen, The Netherlands; 3 Department of General Pediatrics, Heinrich-Heine-University, Düsseldorf, Germany; 4 Microscopical Imaging Centre, Nijmegen Centre for Molecular Life Sciences, Radboud University Nijmegen Medical Centre, Nijmegen, The Netherlands

**Correction to: J Mol Med (2009) 87:515–522**

10.1007/s00109-009-0452-5

We would like to correct the above manuscript as follows:

In Table 1 references 23 and 25 should be replaced by references 19 and 20 (note d), and references 27 and 28 should be replaced by references 23 and 31 (note e). Both notes (d and e) should state that “the values presented were taken from the indicated references as depicted in the caption to Figure 1.”

The sentence: “Linear regression analysis revealed a negative correlation between ROS levels and ∆ψ (Fig. 1b). We have also shown that, similar to cellular ROS levels, ERCa is variably decreased in these patient fibroblasts (Table 1; [19]). Again, a significant, this time positive correlation was observed (Fig. 1c).”

Should be corrected to: “Linear regression analysis revealed a negative correlation between ROS levels and ∆ψ (Fig. 1b; performed on error-weighted means of all TMRM values [3 controls and 10 patients] presented in Table 1 and Figure 1b). We have also shown that, similar to cellular ROS levels, ERCa is variably decreased in these patient fibroblasts (Table 1; [19]). Again, a significant, this time positive correlation was observed (Fig. 1c).”

